# Hierarchical Cluster Analysis of Semicircular Canal and Otolith Deficits in Bilateral Vestibulopathy

**DOI:** 10.3389/fneur.2018.00244

**Published:** 2018-04-10

**Authors:** Alexander A. Tarnutzer, Christopher J. Bockisch, Elena Buffone, Konrad P. Weber

**Affiliations:** ^1^Department of Neurology, University Hospital Zurich, University of Zurich, Zurich, Switzerland; ^2^Department of Ophthalmology, University Hospital Zurich, University of Zurich, Zurich, Switzerland; ^3^Department of Otorhinolaryngology, University Hospital Zurich, University of Zurich, Zurich, Switzerland

**Keywords:** vestibulo-ocular reflex, video-head-impulse testing, Menière’s disease, aminoglycoside-related ototoxicity, vestibular-evoked myogenic potentials

## Abstract

**Background:**

Gait imbalance and oscillopsia are frequent complaints of bilateral vestibular loss (BLV). Video-head-impulse testing (vHIT) of all six semicircular canals (SCCs) has demonstrated varying involvement of the different canals. Sparing of anterior-canal function has been linked to aminoglycoside-related vestibulopathy and Menière’s disease. We hypothesized that utricular and saccular impairment [assessed by vestibular-evoked myogenic potentials (VEMPs)] may be disease-specific also, possibly facilitating the differential diagnosis.

**Methods:**

We searched our vHIT database (*n* = 3,271) for patients with bilaterally impaired SCC function who also received ocular VEMPs (oVEMPs) and cervical VEMPs (cVEMPs) and identified 101 patients. oVEMP/cVEMP latencies above the 95th percentile and peak-to-peak amplitudes below the 5th percentile of normal were considered abnormal. Frequency of impairment of vestibular end organs (horizontal/anterior/posterior SCC, utriculus/sacculus) was analyzed with hierarchical cluster analysis and correlated with the underlying etiology.

**Results:**

Rates of utricular and saccular loss of function were similar (87.1 vs. 78.2%, *p* = 0.136, Fisher’s exact test). oVEMP abnormalities were found more frequent in aminoglycoside-related bilateral vestibular loss (BVL) compared with Menière’s disease (91.7 vs. 54.6%, *p* = 0.039). Hierarchical cluster analysis indicated distinct patterns of vestibular end-organ impairment, showing that the results for the same end-organs on both sides are more similar than to other end-organs. Relative sparing of anterior-canal function was reflected in late merging with the other end-organs, emphasizing their distinct state. An anatomically corresponding pattern of SCC/otolith hypofunction was present in 60.4% (oVEMPs vs. horizontal SCCs), 34.7% (oVEMPs vs. anterior SCCs), and 48.5% (cVEMPs vs. posterior SCCs) of cases. Average (±1 SD) number of damaged sensors was 6.8 ± 2.2 out of 10. Significantly (*p* < 0.001) more sensors were impaired in patients with aminoglycoside-related BVL (8.1 ± 1.2) or inner-ear infections (8.7 ± 1.8) compared with Menière-related BVL (5.5 ± 1.5).

**Discussion:**

Hierarchical cluster analysis may help differentiate characteristic patterns of BVL. With a prevalence of ≈80%, utricular and/or saccular impairment is frequent in BVL. The extent of SCC and otolith impairment was disease-dependent, showing most extensive damage in BVL related to inner-ear infection and aminoglycoside-exposure and more selective impairment in Menière’s disease. Specifically, assessing utricular function may help in the distinction between aminoglycoside-related BVL and bilateral Menière’s disease.

## Introduction

Key complaints in patients with bilateral loss of peripheral-vestibular function [bilateral vestibular loss (BVL)] are unsteadiness of gait (worse in the dark and on uneven surfaces), postural imbalance, blurred vision (i.e., “oscillopsia”) during head movements due to an insufficient angular vestibulo-ocular reflex (aVOR) and impaired spatial orientation (1–6). We previously characterized the distribution of affected semicircular canals (SCCs) in a cohort of 109 patients with BVL due to various causes and described disease-specific patterns of SCC hypofunction (7). Whereas for BVL related to infectious inner-ear disorders, cerebellar ataxia–neuropathy–vestibular areflexia syndrome (CANVAS) and bilateral hearing-loss horizontal, anterior and posterior SCCs were equally affected, we found significant sparing of the anterior SCCs in patients with aminoglycoside-related BVL, bilateral Menière’s disease and idiopathic BVL. While our previous study provided detailed information on SCC function, it did not assess the functional integrity of the otolith organs (i.e., the utriculus and sacculus). Previous studies have indicated impairment of the otolith organs as well in BVL, using eccentric rotation (8) or inter-aural linear head motion (9). These paradigms, however, are limited in their applicability, as they allow testing of utricular function only and provide information on bilateral utricular function only in case of inter-aural accelerations. More detailed and targeted testing of utricular and saccular function became available with the introduction of vestibular-evoked myogenic potentials (VEMPs) (10, 11). Compared with loss of function of the horizontal SCCs, saccular impairment has previously been reported to be less frequent, with unilaterally absent responses on cervical VEMPs (cVEMPs) in 4/84 cases only and no cases with bilaterally absent responses (12). In another study, response amplitudes in BVL patients were below the 5th percentile of values in healthy normal subjects in 64% [ocular VEMPs (oVEMPs)] and 61% (cVEMPs), respectively (13). In their study, utricular function differed significantly depending on the underlying cause of BVL, being worst for aminoglycoside-related vestibulopathy and best for Menière’s disease.

In analogy to the observed disease-specific pattern of SCC function and following-up on previous studies restricted to the assessment of horizontal SCC function, we hypothesized that the pattern of utricular and saccular dysfunction in BVL may be disease-specific and associated with different SCCs. Based on the results published by Agrawal and coworkers (13), we predicted worst utricular function in aminoglycoside-related vestibulopathy. Furthermore, there is currently no convincing explanation for the disease-specific patterns of SCC hypofunction in BVL. Possibly, peripheral-vestibular hypofunction in BLV follows the anatomy of the vestibular nerve or the vestibular artery. Alternatively, other—yet poorly understood—mechanisms affecting SCC and otolith hair cell function such as local inflammation, toxins [see, e.g., Ref. (14)] or disruption of the endolymphatic membrane may lead to BLV. In the first case, we predict (a) simultaneous normal or impaired functioning of the anterior and horizontal canal and the utriculus (being supported by the superior branch of vestibular nerve/artery) and (b) normal or impaired functioning of the posterior SCC and the sacculus (being linked to the inferior branch of vestibular nerve/artery) (15). In the latter case (i.e., damage to the hair cells), no such correlation is expected. To address these predictions in patients with BVL, we analyzed results from video-head-impulse testing (vHIT) of all six SCCs and VEMPs of both the utriculus (termed oVEMPs) and the sacculus (termed cVEMPs) with hierarchical cluster analysis and correlated the pattern of SCC, utricular and saccular hypofunction with the underlying cause of BVL.

## Materials and Methods

This study was carried out in accordance with the recommendations of the Cantonal Ethics Committee Zurich and in accordance with the Declaration of Helsinki. As this was a retrospective database analysis, retrieval of informed written consent from all involved patients was not feasible. The protocol was approved by the Cantonal Ethics Committee Zurich and exempt for retrieval of written informed consent was granted (study protocol 2013-0468). We retrospectively screened our vHIT database for patients with a diagnosis of BVL, i.e., that demonstrated vestibular loss in at least one SCC on each side, that have also received otolith testing. This search was last updated in March 2016 and included the entire time period after introducing testing of both the horizontal and vertical SCCs at our clinic in October 2012.

### vHIT Recording Procedure

The standard procedure used for testing individual SCCs by vHIT at our clinic requires 20 valid head impulses for each canal [see Ref. (16) for a detailed description], with SCCs tested in pairs according to the planes of stimulation (horizontal canals, RALP plane for right anterior and left posterior canal, LARP plane for left anterior and right posterior canal). For video-oculography, commercially available video-head-impulse testing goggles (Otometrics, Taastrup, Denmark) with an infrared camera recording the right eye was used. Horizontal and vertical eye position was measured at a frequency of 250 Hz, and angular head velocity was determined by three orthogonal mini-gyroscopes. For further analysis, eye and head velocity values were calculated.

### VEMP Recording Procedure

We reviewed otolith function as assessed by cVEMPs (saccular testing, air- or bone-conducted stimulation) and oVEMPs (utricular testing, bone-conducted stimulation). Calibrated headphones (Telephonics TDH-39P; Telephonics Corp., Farmingdale, NY, USA) were used to apply air-conducted sound stimuli (500 Hz, 6 ms tone bursts at 90–100 dB normal hearing level, total of 200 bursts) monaurally to the right and left ears for cVEMPs. The procedure was identical to the one described by Blanquet and colleagues (17): “During stimulation, subjects were asked to sit and turn their head as much as possible to the side to tense their sternocleidomastoid muscle (SCM). EMG activity was recorded (Viking V system; Nicolet Biomedical, Madison, WI, USA) from the upper half of the SCM ipsilateral to the side of acoustic stimulation. A reference electrode was placed on the upper part of the sternum. The background SCM contraction was monitored online and measured over the 20-ms prestimulus interval (using root-mean-square EMG amplitude). Signals of 200 air-conducted cVEMP stimuli were averaged, as previously reported by Poretti et al. (18). Note that in case of inconclusive or negative air-conducted cVEMPs, bone-conducted cVEMPs were obtained and judgment was based on the findings from the latter. Vibrations (unshaped 500 Hz bursts resulting in inter-aural accelerations of about 0.1 g, duration 4 ms, 200 stimuli in total) were applied using a Minishaker (Model 4810, Brüel & Kjaer, P/L, Naerum, Denmark) placed over the hairline near Fz, as previously described by Weber et al. (19). Again, responses from the contralateral SCM were recorded. To improve reproducibility of measurements and to reduce noise from asymmetric muscle tension in individuals, response amplitudes were normalized. This procedure is based on the assumption that there is a linear relationship between the level of muscle contraction and the response amplitude. This was demonstrated for moderate to strong muscle contractions (20) and confirmed by Rosengren more recently (21). Reported values for air- and bone-conducted cVEMPS will therefore be unitless.” Only responses obtained at the highest stimulus intensity applied were considered. If more than one measurement was obtained at this intensity, we calculated the average.

“Bone-conducted oVEMPs (unshaped 500 Hz bursts resulting in inter-aural accelerations of about 0.1 g, duration 4 ms, 200 stimuli in total) were applied by the same Minishaker, placed again over the hairline near Fz. Stimuli were recorded with surface electrodes placed beneath the eyes during up-gaze,” as described by Blanquet and colleagues (17). Further details can be found here (10, 11).

### Patient Identification and Data Analysis

Previously, we have reported on patterns of SCC hypofunction in patients with vHIT-confirmed BVL (7). If serial vHITs were obtained in individual patients and concomitant VEMPs were available only from one session, all results were selected from this session. We re-analyzed aVOR gains in all patients using Otosuite Version 3.0 (Otometrics, Taastrup, Denmark) and used custom-written MATLAB (R2017b, The MathWorks, Natick, MA, USA) routines for the quantification of corrective saccades. This provided cumulative overt saccade amplitudes [for detailed analysis, see Ref. (7)]. Vestibular hypofunction was defined as a reduction in VOR gain and/or the occurrence of compensatory saccades. For a diagnosis of BVL, hypofunction of at least one canal on either side was required; importantly, these two canals could be coplanar or not. For gains, cutoff values of 0.8 (for the horizontal canals) and 0.7 (for the vertical canals) have been proposed by the manufacturer of the video-goggles (GN otometrics) to distinguish normal from reduced aVOR function. Recently proposed cutoff values suggest that cumulative saccade amplitudes above 0.7–0.8°/trial indicate loss of function of the canal tested (7, 22). Here, we adhered to the cutoff value (0.73°/trial) proposed by Tarnutzer et al. (7), as the same statistical approach was used. The underlying cause of BVL—if identified—was retrieved from the patients’ clinical files. Files were screened for exposure to vestibulotoxic drugs such as aminoglycosides and to CNS infections. We followed the AAO-HNS 1995 guidelines for diagnosing MD (23). A diagnosis of bilateral sensorineural hearing loss (SNHL) required documented hearing impairment as assessed by pure tone audiogram based on CPT-AMA guidelines (24) with a CPT value > 20% on both sides and exclusion for other causes. Diagnostic criteria for CANVAS were based on the definition provided by Szmulewicz et al. (25). MR imaging was required to confirm vestibular schwannoma or central causes. The diagnosis of vestibular neuritis was based on clinical grounds (defined as a single episode with acute-onset, prolonged vertigo or dizziness and spontaneous nystagmus) as documented in the patient’s medical records and—if available—on vestibular testing in the acute stage (26).

Two experienced neurootologists (Alexander A. Tarnutzer and Konrad P. Weber) independently reviewed all vHIT traces. Interrater agreement for individual canal function (normal vs. pathological) was 0.85 (Cohen’s kappa) (27). Traces were evaluated for reduced aVOR gain, increased corrective saccades or a combination of both (7). Discordant ratings were resolved by discussion among the two reviewers.

For assessing the integrity of the utriculus and sacculus, three different VEMP parameters were included: peak-to-peak amplitudes, amplitude asymmetry ratio (left vs. right side) and latencies. Left to right amplitude asymmetries of more than 30% were considered abnormal for oVEMPs and cVEMPs. This was based both on normative values obtained with the same setup and derived cutoff values (defined as mean + 2 SD) and the range of cutoff values typically proposed in the literature (10). Regarding response latencies and peak-to-peak amplitudes, values were compared with those recorded from 26 healthy human subjects (aged 38.4 ± 15.8 years; 11 females) with the same setup. For latency, values above the 95th percentile of values in the controls were considered abnormal, while for amplitudes, values below the 5th percentile of normal values were defined as abnormal (for details see Table 1).

**Table 1:** Diagnostic criteria for impairment of the semicircular canals (SCCs) and the otolith organs.

SCCs	Method: vHIT Parameters: Gain Catch-up saccades Definition of impairment (at least one): Reduced gains (horizontal canals <0.8; vertical canals <0.7) Overt/covert catch-up saccades
Utriculus	Method: bone-conducted oVEMPsParameters: Peak-to-peak amplitude (n10–p15) Amplitude asymmetry (L/R) Response latencies (n10, p15) Definition of impairment (at least one) Amplitude < 5.8 μV Amplitude asymmetry > 30% Response latencies > 95th percentile of normal: n10 > 13.8 ms p15 > 18.7 ms
Sacculus	Method: air-conducted/bone-conducted cVEMPsParameters: 4. Peak-to-peak amplitude (p13–n23) 5. Amplitude asymmetry (L/R) 6. Response latencies (p13, n23) Definition of impairment (at least one) Amplitude < 0.8^a^ Amplitude asymmetry > 30% Response latencies > 95th percentile of normal: p13 > 17.3 ms n23 > 30.3 ms

*^a^Peak-to-peak amplitudes for air- and bone-conducted cVEMPs were normalized for sternocleidomastoid muscle contraction level. Therefore, these values are unitless*.

*cVEMPs, cervical vestibular-evoked myogenic potentials; L, left; oVEMPs, ocular vestibular-evoked myogenic potentials; R, right; vHIT, video-head-impulse testing*.

Individual patterns of SCC and otolith hypofunction were analyzed and compared for different underlying disorders. MATLAB and SPSS 23 (IBM, Armonk, NY, USA) were used for statistical analyses. Fisher’s exact test with Bonferroni correction for multiple tests was applied to determine significant differences in the frequency of specific conditions (such as impaired vs. normal peripheral-vestibular function). Analysis of gain values and cumulative saccade amplitudes was based on non-parametric analysis of variance (Kruskal–Wallis ANOVA) with Tukey–Kramer correction for multiple tests. The level of significance for all statistical tests was *p* = 0.05. We applied a generalized linear model (SPSS) to analyze effects of the underlying disorders on the extent of peripheral-vestibular impairment. Fisher’s least significant difference method was used to correct for multiple comparisons when performing pairwise comparisons between the different diagnoses.

For visualization of coherent patterns in large data sets the cluster heat map has been very useful and became one of the most popular graphical illustrations in biological sciences (28). We implemented this approach to our data analysis to identify patterns of vestibular impairment in patients with bilateral vestibulopathy of diverse origin. For each sensor and subject, the functional state (based on the overall rating) was retrieved (intact vs. deficient). We applied hierarchical clustering using the clustergram function (MATLAB) to obtain heat maps with dendrograms of the entire data set [see Ref. (29)]. The heat map was clustered by Euclidean distance, i.e., the geometric distance of the single (raw) data points in the multidimensional space. The data were standardized along the data columns, i.e., for the individual results from single subjects. This means that values are transformed such that the mean is 0 and the SD is 1 in the specified dimension (i.e., each column—reflecting the results from a single subject—in our analysis). This standardized value will then be depicted in a range of colors between 2.5 (dark blue) to −2.5 (dark red) as indicated by the legend. Note, however, that for individual patients, only one intensity of blue and red (being more or less dark/light) are used as only two functional states (1 = intact, 0 = deficient) are possible. The intensity of the colors depends on the calculated SD when performing the standardization. If all 10 sensors had the same functional state (i.e., were deficient) in a single patient, resulting values after standardization in these cases will be 0 and by definition coded by white color. Cluster dendrograms in our data set indicate those patients (*x*-axis) and vestibular sensors (*y*-axis) that are the least different, as these groups cluster together first. More distinct clusters will group later.

## Results

From the 3,271 patients stored in the vHIT database, 142 patients with suspected BVL were identified and their vHIT recordings were reviewed independently by two reviewers (Alexander A. Tarnutzer and Konrad P. Weber). Eventually, 101 patients with confirmed BVL who also had received quantitative testing of both the utricles and the saccules were included (38 females and 63 males, 61.9 ± 16.8 years old, mean age ± 1 SD). Findings of 68 of those 101 patients were previously reported in a study restricted to video-head-impulse testing (7). A diagnosis of the underlying cause of BVL could be identified in 47/101 cases (46.6%), with vestibulotoxic drugs (12/101, 11.8%), Menière’s disease (11/101, 10.9%), and infections involving the inner ear (7/101, 6.9%) being the most frequent disorders (see Table 2 for details).

**Table 2:** Epidemiological findings of the 101 patients with BVL and both SCC and otolith testing available.

Disease	Cases (%)
Unclear	54 (53.4)
Vestibulotoxic drugs^c^	12 (11.8)
Menière’s disease	11 (10.9)
Infectious	7 (6.9)
Bilateral SNHL	3 (3.0)
CANVAS	2 (2.0)
Autoimmune^a^	3 (3.0)
Head trauma	2 (2.0)
Bilateral schwannoma	4 (4.0)
Central causes^b^	2 (2.0)
Schwannoma + VN^d^	1 (1.0)
Total	101 (100)

*BVL, bilateral vestibular loss; CANVAS, cerebellar ataxia, neuropathy, vestibular areflexia syndrome; SCC, semicircular canal; SNHL, sensorineural hearing loss; VN, vestibular neuritis*.

*^a^One case of possible Cogan’s syndrome, one case with Wegener granulomatosis, one case with unknown autoimmune-related disorder*.

*^b^MRI-confirmed cavernoma in the left brachium pontis with possible involvement of the vestibular nuclei in one case, cerebellar ataxia with bilateral vestibulopathy (but no signs of ganglionopathy or polyneuropathy) in the other case*.

*^c^This includes the following aminoglycosides: gentamicin (*n* = 9), tobramicin (*n* = 2). In one case, the type of aminoglycoside remained unclear*.

*^d^Sequential occurrence of vestibular schwannoma on one side and vestibular neuritis on the other side (*n* = 1)*.

**Figure 1:**
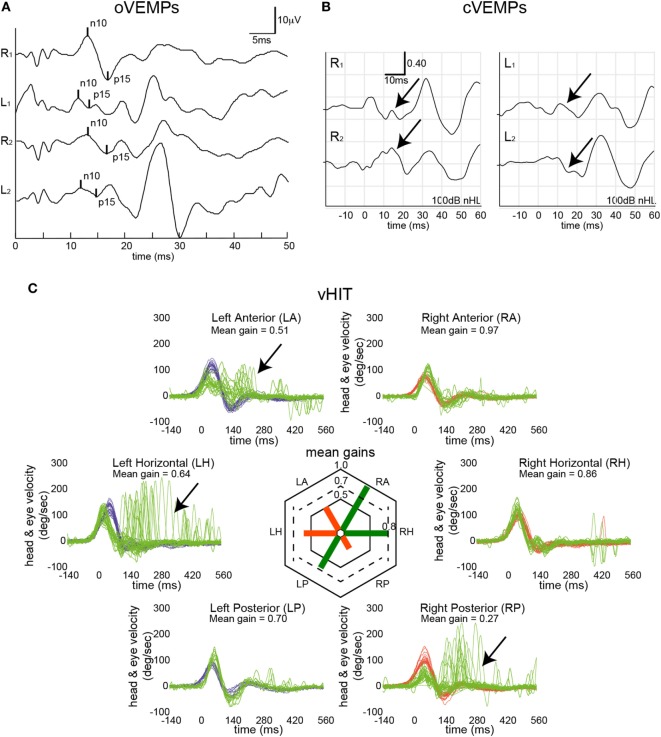
Vestibular mapping in a single patient with bilateral Menière’s disease. Otolith testing indicated left-sided utricular impairment [ocular vestibular-evoked myogenic potentials (oVEMPs) **(A)**] based on two repetitions with an average asymmetry ratio of 67% (cutoff ≤ 30%) and a reduced peak-to-peak amplitude (3.64 µV, 5th percentile = 5.8 μV), while n10 and p15 latencies were within normal range. Bone-conducted cervical vestibular-evoked myogenic potentials (cVEMPs) **(B)** were bilaterally absent (as indicated by the black arrows). In panel **(C)**, eye velocity traces (in green) and head velocity traces (in blue for head turns to the left and in red for head turns to the right) are plotted against time for each semicircular canal (20 trials per canal recorded). Note that eye velocity traces were inverted for better visualization and comparison with the head velocity traces. Catch-up saccades identified by OtosuiteV 3.0 are presented in dark red. vHIT demonstrated loss of function (as indicated by the black arrows) in the left horizontal and left anterior canal and the right posterior canal.

Single subject data are shown in Figure 1 for a patient with bilateral Menière’s disease. While on the left side the horizontal and the anterior canal were impaired, it was the posterior canal on the right side that demonstrated reduced gain and correction saccades. In addition, oVEMPs indicated impaired utricular function on the left side, while cVEMPs suggested bilateral saccular loss of function.

### Distribution of Affected SCCs, Gains and Cumulative Saccade Amplitudes

**Figure 2:**
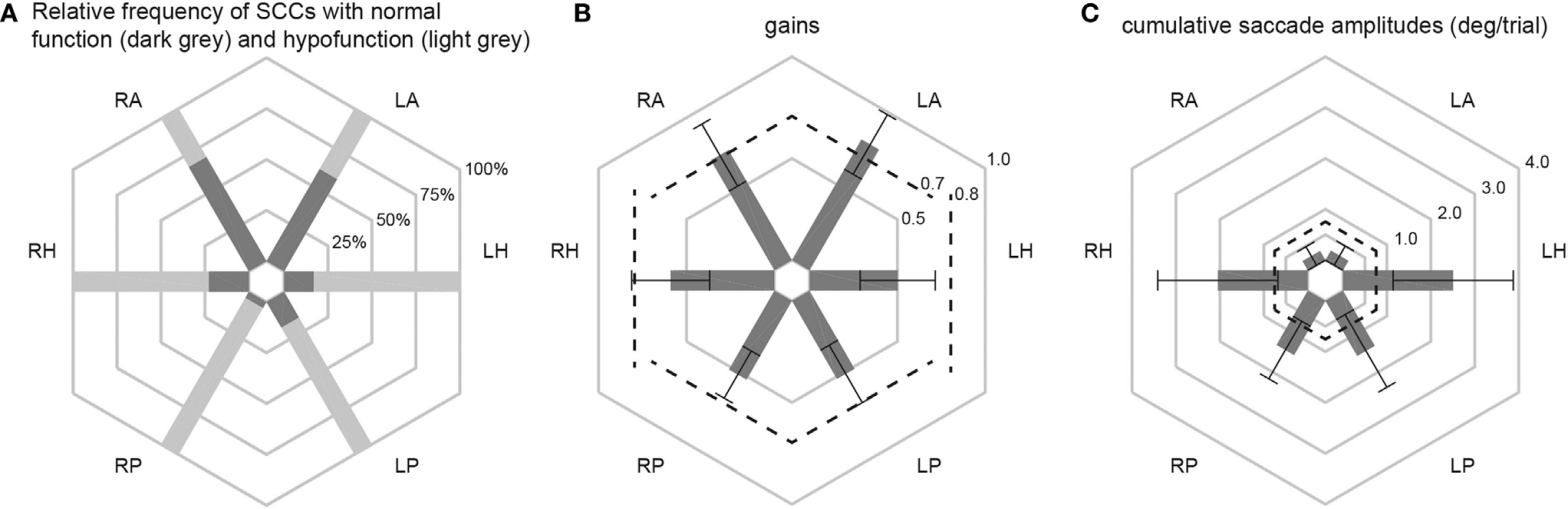
In panel **(A)**, the percentage of patients with normal function (dark gray shaded areas) and with hypofunction (light gray shaded areas) for the different semicircular canals (SCCs) are illustrated in a hexplot. Median (±1 median absolute deviation) gains **(B)** and cumulative saccadic amplitudes **(C)** of all patients (*n* = 101) are shown separately. Gain values (from 0 to 1) and cumulative saccadic amplitudes (°/trial, from 0 to 4) are provided along the different hexagons. Cutoff values for reduced gains (<0.8 for the horizontal canals, <0.7 for the vertical canals) and for abnormally increased cumulative saccade amplitudes (>0.73°/trial) are indicated by dashed lines.

Different aspects of peripheral-vestibular loss are illustrated in this section. This includes statistical analysis of the frequency of SCCs rated as having peripheral-vestibular hypofunction, distribution of gain values and cumulative saccade amplitudes (see Figure 2; Table 3). Similar as in our previous publication (7), no significant (*p* > 0.05, Fisher’s exact test) left/right differences were found regarding the frequency of SCCs rated as deficient. Therefore, trials from the left and right side were pooled for further analyses. This was also true for the distribution of gains and cumulative saccade amplitudes.

**Table 3:** SCC function—overall rating, gains and saccade amplitudes.^a^

Group	Fraction of SCCs with rated hypofunction (%)				Median gains (1 MAD)				Median cumulative saccade amplitudes (1 MAD) (°/trial)			
			
	Hor	Ant	Post	Stats	Hor	Ant	Post	Stats	Hor	Ant	Post	Stats
All (*n* = 101)	162/202 (80.2%)	72/202 (35.6%)	181/202 (89.6%)	H vs. A: *p* < 0.001* H vs. P: *p* = 0.036* P vs. A: *p* < 0.001*	0.56 (0.23)	0.76 (0.21)	0.52 (0.17)	H vs. A: *p* < 0.001* H vs. P: *p* = 0.052 P vs. A: *p* < 0.001*	2.29 (1.50)	0.25 (0.25)	1.44 (0.82)	H vs. A: *p* < 0.001* H vs. P: *p* = 0.002* P vs. A: *p* < 0.001*

Unclear (*n* = 54)	86/108 (79.6%)	33/108 (30.6%)	96/108 (88.9%)	H vs. A: *p* < 0.001* H vs. P: *p* = 0.275 A vs. P: *p* < 0.001*	0.59 (0.21)	0.78 (0.19)	0.54 (0.15)	H vs. A: *p* < 0.001* H vs. P: *p* = 0.124 P vs. A: *p* < 0.001*	2.04 (1.31)	0.21 (0.21)	1.24 (0.85)	H vs. A: *p* < 0.001* H vs. P: *p* = 0.019* P vs. A: *p* < 0.001*

Vestibulotox. drugs (*n* = 12)	22/24 (91.7%)	8/24 (33.3%)	24/24 (100%)	H vs. A: *p* < 0.001* H vs. P: *p* = 0.578 A vs. P: *p* < 0.001*	0.45 (0.17)	0.75 (0.14)	0.42 (0.10)	H vs. A: *p* = 0.018* H vs. P: *p* = 0.993 P vs. A: *p* = 0.013*	2.85 (1.10)	0.38 (0.30)	1.85 (0.43)	H vs. A: *p* < 0.001* H vs. P: *p* = 0.546 P vs. A: *p* = 0.003*

Menière’s disease (*n* = 11)	13/22 (59.1%)	3/22 (13.6%)	17/22 (77.3%)	H vs. A: *p* = 0.012* H vs. P: *p* = 0.332 A vs. P: *p* < 0.001*	0.77 (0.12)	0.99 (0.11)	0.61 (0.11)	H vs. A: *p* = 0.011* H vs. P: *p* = 0.235 P vs. A: *p* < 0.001*	1.37 (1.05)	0.01 (0.01)	1.16 (0.87)	H vs. A: *p* < 0.000* H vs. P: *p* = 0.489 P vs. A: *p* = 0.001*

Infectious (*n* = 7)	13/14 (92.9%)	10/14 (71.4%)	14/14 (100%)	H vs. A: *p* = 0.328 H vs. P: *p* = 1.000 A vs. P: *p* = 0.105	0.32 (0.14)	0.44 (0.26)	0.26 (0.08)	H vs. A: *p* = 0.381 H vs. P: *p* = 0.915 P vs. A: *p* = 0.196	4.24 (0.88)	1.74 (0.72)	2.81 (1.31)	H vs. A: *p* = 0.009* H vs. P: *p* = 0.185 P vs. A: *p* = 0.452

*A/Ant, anterior; H/Hor, horizontal; MAD, median absolute deviation; P/Post, posterior; SCC, semicircular canal*.

*Indicate statistically significant i.e. (*p* < 0.05) differences.

*^a^Since statistical analysis showed no effects of laterality (*p* > 0.05), results from left and right sides were pooled for further analyses*.

Overall, fractions of SCCs rated as having peripheral-vestibular hypofunction were significantly (*p* < 0.001) larger for the horizontal and posterior SCCs than for the anterior SCCs. Likewise, compared with the posterior and horizontal SCCs, anterior SCCs showed significantly (*p* < 0.001) higher median gains and significantly (*p* < 0.001) smaller cumulative saccade amplitudes. In agreement with our previous publication, which included subpopulations with at least five samples studied with greater detail, rates of affected SCCs were not significantly different (*p* > 0.05) for infectious inner-ear disorders. At the same time, we noted significantly higher rates of impairment for the horizontal and posterior canals compared with the anterior canals in patients with aminoglycoside-induced BVL, Menière’s disease and idiopathic causes (see Table 3 for specific *p*-values).

In 12 patients (11.9%), vHIT demonstrated bilaterally normal horizontal canal function. In 10 out of those 12 patients, the posterior canals were bilaterally impaired, with SCC impairments being restricted to the posterior canals in 8/12. In these patients, cVEMPs were more frequently abnormal than oVEMPs (75%; bilateral in 4 and unilateral in 5) vs. 50% (6/12; bilateral in 2, unilateral in 4). Underlying diagnoses in these patients were BVL of unclear origin (*n* = 6), bilateral Menière’s disease (*n* = 4), bilateral SNHL (*n* = 1), and bilateral schwannoma (*n* = 1).

### oVEMPs—Response Latencies and Amplitudes

Median [±1 median absolute deviation (MAD)] latencies in oVEMPs were calculated for the n10 and p15 response and peak-to-peak amplitude (n10–p15) was determined, with values from both sides pooled for calculation, as they did not differ significantly (*p* > 0.05, *t*-test). oVEMP responses were unilaterally (*n* = 15) or bilaterally (*n* = 33) absent in 48 patients. In those with preserved oVEMP responses, n10 latencies were above the 95th percentile of latencies in healthy controls in 22 patients (unilateral = 15, bilateral = 7). Overall, abnormal (i.e., either delayed or absent) n10 responses (unilateral or bilateral) were found in 66.3% (67/101) of all patients. Likewise, in those with preserved oVEMP responses, p15 latencies were above the 95th percentile of latencies in healthy controls in 12 patients (unilateral = 10, bilateral = 2). Overall, abnormal (i.e., either delayed or absent) p15 responses (unilateral or bilateral) were found in 58.4% (59/101) of all patients (see Table 4 for details).

**Table 4:** Ocular vestibular-evoked myogenic potential amplitudes and latencies.

	Peak-to-peak amplitude^a^			n10 Latency^a^			p15 Latency^a^		
			
	<5th % unilateral (%)^b^	<5th % bilateral (%)^b^	<5th % total (%)^b^	>95th % unilateral (%)^c^	>95th % bilateral (%)^c^	>95th % total (%)^c^	>95th % unilateral (%)^c^	>95th % bilateral (%)^c^	>95th % total (%)^c^
Unclear (*n* = 54)	17/54 (31.5)	20/54 (37.0)	37/54 (68.5)	13/54 (24.1)	22/54 (40.7)	35/54 (64.8)	13/54 (24.1)	17/54 (31.5)	30/54 (55.6)
Vestibulotoxic drugs (*n* = 12)	2/11 (26.7)	9/12 (75.0)	11/12 (91.7)	2/12 (26.7)	9/12 (75.0)	11/12 (91.7)	3/12 (25)	8/12 (66.7)	11/12 (91.7)
Menière’s disease (*n* = 11)	2/11 (18.2)	4/11 (36.4)	6/11 (54.6)	3/11 (27.3)	3/11 (27.3)	6/11 (54.6)	1/11 (9.1)	2/11 (18.2)	3/11 (27.3)
Infectious (*n* = 7)	2/7 (28.6)	5/7 (71.4)	7/7 (100.0)	1/7 (14.3)	5/7 (71.4)	6/7 (85.7)	1/7 (14.3)	5/7 (71.4)	6/7 (85.7)
All (*n* = 101)	32/101 (31.6)	45/101 (44.6)	77/101 (76.2)	24/101 (23.7)	43/101 (42.6)	67/101 (66.3)	23/101 (22.8)	36/101 (35.6)	59/101 (58.4)

*^a^Since statistical analysis showed no effects of laterality (*p* > 0.05), results from left and right sides were pooled for further analyses. This includes those patients with preserved but abnormal (i.e., with increased latency or decreased amplitude) responses and those with absent responses*.

*^b^Significant reductions in peak-to-peak amplitude were defined as amplitudes below the 5th percentile of peak-to-peak amplitudes in the healthy controls (5th percentile = 5.8 μV)*.

*^c^Significant increases in latency were defined as latencies above the 95th percentile of latency-values in the healthy controls (n10 = 13.8 ms; p15 = 18.7 ms)*.

**Figure 3:**
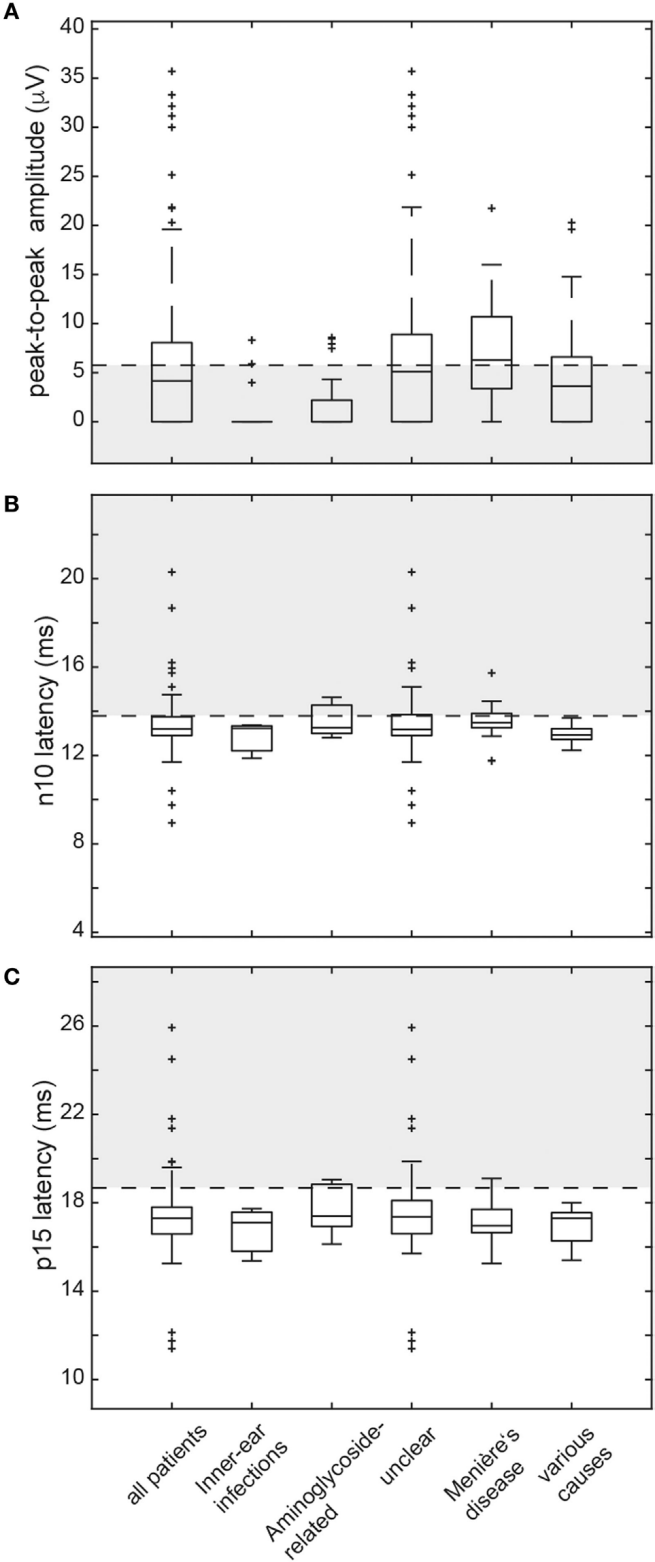
Box and whisker plots illustrating both peak-to-peak amplitudes (μV) and latencies (ms) for ocular vestibular-evoked myogenic potentials (oVEMPs) in all patients. For peak-to-peak (n10–p15) amplitudes **(A)**, a horizontal dashed line refers to the lower limit of normal (i.e., the 5th percentile of amplitudes measured in the healthy controls). The gray shaded area below indicates significantly reduced amplitudes. For both n10 latencies **(B)** and p15 latencies **(C)**, the upper limit of normal (i.e., the 95th percentile of latency values in the healthy controls) is indicated by a horizontal dashed line. The gray shaded area above refers to significantly increased latencies. The box has lines at the lower quartile, the median, and the upper quartile values. Whiskers extend from each end of the box to 1.5 times the interquartile range from the ends of the box. Outliers (black “+” sign) are data with values beyond the ends of the whiskers. Note that subjects with absent oVEMP responses are not shown on this figure.

In those patients with preserved oVEMP responses, amplitudes were below the 5th percentile of responses in healthy controls in 34 (unilateral = 27, bilateral = 7). Overall, amplitudes were (unilateral or bilateral) abnormal (i.e., either below the 5th percentile or absent) in 76.2% (77/101) of all patients. Rates of reduced amplitudes in our patients were significantly higher than rates of increased n10 (*p* = 0.010) or p15 (*p* < 0.001) latencies. The distribution of amplitudes and oVEMP latencies is illustrated in Figure 3 both for the entire study population and the most frequent diagnoses. Overall, rates of abnormally increased latencies (n10, p15) and reduced amplitudes were highest for those patients with aminoglycoside-related BVL and those with a history of inner-ear infections, whereas they were lower for Menière’s disease and in those cases with BVL of unclear origin (Table 4). The peak-to-peak oVEMP amplitude asymmetry ratio (left side vs. right side) was calculated in patients that had preserved responses on at least one side (*n* = 68). Significant asymmetry ratios (i.e., >30%) were identified in 35 patients.

The number of parameters showing impairment (n10 latency, p15 latency, peak-to-peak amplitude, amplitude asymmetry) differed significantly among the subgroups (df = 4, chi-square = 15.777, *p* = 0.003; generalized linear model). Pairwise comparisons demonstrated higher average numbers of parameters affected in patients with BVD related to inner-ear-infections or status post aminoglycoside treatment compared with those with Menière’s disease, various causes and unclear origin (*p* ≤ 0.042).

When pooling the different parameters indicating utricular hypofunction, abnormalities were noted in 88/101 patients (bilateral = 57, unilateral = 31). Among the different subgroups with specific diagnoses (i.e., BVL related to inner-ear infections, aminoglycosides, Menière’s disease, unclear causes), utricular impairment (both sides pooled) was significantly more frequent in aminoglycoside-related BVL compared with bilateral Menière’s disease (91.7 vs. 54.6%, *p* = 0.039, Fisher’s exact test, Bonferroni-corrected for multiple comparisons). Among the other subgroups, no significant differences were found.

### cVEMPs—Response Latencies and Amplitudes

Median (±1 MAD) latencies were determined for the p13 and the n23 responses and peak-to-peak amplitudes (p13–n23) were calculated. Again, values from both sides were pooled, as they did not differ significantly (*p* > 0.05, *t*-test). cVEMP responses were unilaterally (*n* = 14) or bilaterally (*n* = 24) absent in 38 patients. In those patients with preserved cVEMP responses, p13 latencies were above the 95th percentile of latencies in healthy controls in 11 (unilateral = 8, bilateral = 3). Overall, abnormal (i.e., either delayed or absent) p13 responses (unilateral or bilateral) were found in 47.5% (48/101) of all patients. Likewise, in those patients with preserved cVEMP responses, n23 latencies were above the 95th percentile of latencies in healthy controls in 4 (unilateral = 4, bilateral = 0). Overall, abnormal (i.e., either delayed or absent) n23 responses (unilateral or bilateral) were found in 41.6% (42/101) of all patients (see Table 5 for details).

**Table 5:** cVEMP amplitudes and latencies.

	Peak-to-peak amplitude^a^			p13 Latency^a^			n23 Latency^a^		
			
	<5th % unilateral (%)^b^	<5th % bilateral (%)^b^	<5th % total (%)^b^	>95th % unilateral (%)^c^	>95th % bilateral (%)^c^	>95th % total (%)^c^	>95th % unilateral (%)^c^	>95th % bilateral (%)^c^	>95th % total (%)^c^
Unclear (*n* = 54)	19/54 (35.2)	17/54 (31.5)	36/54 (66.7)	8/54 (14.7)	10/54 (18.5)	18/54 (33.3)	5/54 (9.3)	8/54 (14.8)	13/54 (24.1)
Vestibulotoxic drugs (*n* = 12)	1/12 (8.5)	10/12 (83.3)	11/12 (91.7)	3/12 (25.0)	5/12 (41.7)	8/12 (66.7)	3/12 (25.0)	5/12 (41.7)	8/12 (66.7)
Menière’s disease (*n* = 11)	2/11 (17.7)	6/11 (54.6)	8/11 (72.3)	2/11 (18.2)	4/11 (36.4)	6/11 (54.6)	2/11 (18.2)	3/11 (27.3)	5/11 (45.5)
Infectious (*n* = 7)	1/7 (14.3)	5/7 (71.4)	6/7 (85.7)	2/7 (28.6)	5/7 (71.4)	7/7 (100.0)	1/7 (14.3)	5/7 (71.4)	6/7 (85.7)
All (*n* = 101)	25/101 (24.8)	49/101 (48.5)	74/101 (73.3)	20/101 (19.8)	28/101 (27.7)	48/101 (47.5)	18/101 (17.8)	24/101 (23.8)	42/101 (41.6)

*^a^Since statistical analysis showed no effects of laterality (*p* > 0.05), results from left and right sides were pooled for further analyses*.

*^b^Significant reductions in peak-to-peak amplitude were defined as amplitudes below the 5th percentile of peak-to-peak amplitudes in the healthy controls (5th percentile = 0.8). Note that peak-to-peak amplitudes for air- and bone-conducted cervical vestibular-evoked myogenic potentials (cVEMPs) were normalized for sternocleidomastoid muscle contraction level. Therefore, these values are unitless*.

*^c^Significant increases in latency were defined as latencies above the 95th percentile of latency values in the healthy controls (n13 = 17.3 ms; p23 = 30.3 ms)*.

**Figure 4:**
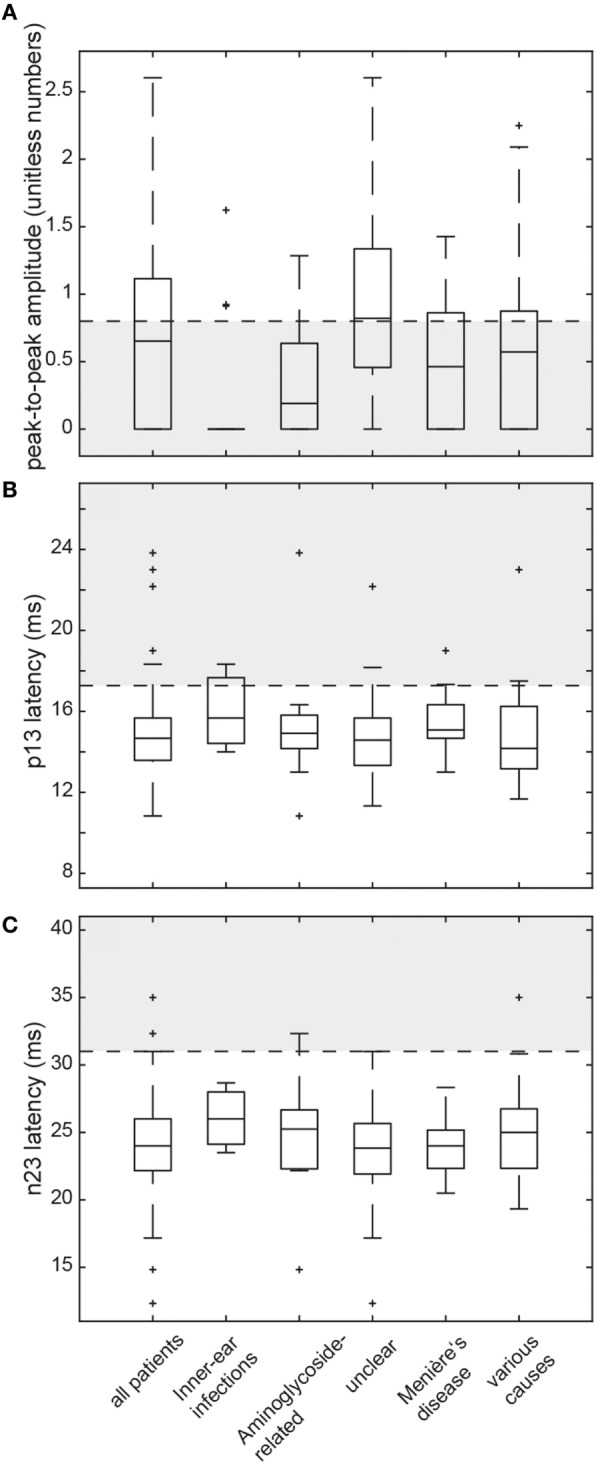
Box and whisker plots illustrating both peak-to-peak amplitudes (unitless numbers) and latencies (ms) for cervical vestibular-evoked myogenic potentials (cVEMPs) in all patients. For peak-to-peak (p13–n23) amplitudes **(A)**, a horizontal dashed line refers to the lower limit or normal (i.e., the 5th percentile of amplitudes measured in the healthy controls). The gray shaded area below indicates significantly reduced amplitudes. For both p13 latencies **(B)** and n23 latencies **(C)**, the upper limit of normal (i.e., the 95th percentile of latency values in the healthy controls) is indicated by a horizontal dashed line. The gray shaded area above refers to significantly increased latencies. For a detailed explanation of the boxes and whiskers, see figure legend of Figure 3. Note that subjects with absent cVEMP responses are not shown on this figure.

In those patients with preserved cVEMP responses, amplitudes were below the 5th percentile of responses in healthy controls in 46 (unilateral = 31, bilateral = 15). Overall, amplitudes were (unilateral or bilateral) abnormal (i.e., either below the 5th percentile or absent) in 73.3% (74/101) of all patients. Rates of reduced amplitudes in our patients were significantly higher than rates of increased p13 (*p* < 0.001) or n23 (*p* < 0.001) latencies. The distribution of amplitudes and cVEMP latencies are illustrated in Figure 4. Overall, rates of abnormally increased latencies (p13, n23) and reduced amplitudes were highest for those patients with aminoglycoside-related BVL and those with a history of inner-ear infections, whereas they were lower for those with BVL of unclear origin (see Table 5). The peak-to-peak amplitude asymmetry ratio was calculated in patients that had preserved oVEMP responses on at least one side (*n* = 78). Significant asymmetry ratios (i.e., >30%) were identified in 26 patients.

The number of parameters showing impairment (p13 latency, n23 latency, peak-to-peak amplitude, amplitude asymmetry) differed significantly among the subgroups (df = 4, chi-square = 20.174, *p* < 0.001). Pairwise comparisons demonstrated higher average numbers of parameters affected in those patients with BVD related to inner-ear-infections compared with those with Menière’s disease, various causes and unclear origin (*p* ≤ 0.035).

Abnormalities in absolute amplitudes, amplitude asymmetries or latencies for cVEMPs could be found in 79/101 patients (bilateral = 50, unilateral = 29). Comparing the frequency of cVEMP impairment (both sides pooled) among the different subgroups with specific diagnoses, we found a significantly higher rate in aminoglycoside-related BVL compared with those cases with BVL of unclear origin (87.5 vs. 50.0%, *p* = 0.004). Among the other subgroups, no significant differences were found.

### Individual Patterns of Utricular and Saccular Impairment

The distribution of VEMP patterns in all patients is illustrated in Table 6, showing various combinations of unilateral or bilateral utricular and/or saccular hypofunction. Most frequently, oVEMPs and cVEMPs were bilaterally (31%) or unilaterally (12%) abnormal, or bilaterally abnormal oVEMPs were accompanied by unilaterally abnormal cVEMPs (16%). Overall, rates of utricular and saccular loss of function (unilateral or bilateral) in the study population (*n* = 101) were not significantly different (87.1 vs. 78.2%, *p* = 0.136). Noteworthy, the lower rates of otolith (saccular or utricular) hypofunction compared with SCC hypofunction (in 100% of cases) were a consequence of the inclusion criteria (bilateral SCC hypofunction) applied here.

**Table 6:** Distribution of utricular and saccular function in the bilateral vestibular loss patients (*n* = 101).

Combination	Fraction (%)
Ocular vestibular-evoked myogenic potentials (oVEMPs) and cervical vestibular-evoked myogenic potentials (cVEMPs) bilaterally abnormal	31
oVEMPs bilaterally abnormal, cVEMPs unilaterally abnormal,	16
cVEMPs and oVEMPs unilaterally abnormal	12
cVEMPs bilaterally abnormal, oVEMPs unilaterally abnormal	11
cVEMPs bilaterally normal, oVEMPs bilaterally abnormal	10
cVEMPs bilaterally abnormal, oVEMPs bilaterally normal	8
cVEMPs bilaterally normal, oVEMPs unilaterally abnormal	8
cVEMPs and oVEMPs bilaterally normal	4
cVEMPs unilaterally abnormal, oVEMPs bilaterally normal	<1

### Comparison Between Otolith Function and SCC Function

We compared overall otolith function (VEMPs) and SCC function (vHIT), looking at joint probabilities. This analysis was driven by the question whether the patterns of vestibular (SCC and/or otolith) impairment in BVL match the anatomy of the vascular supply and the innervation of the vestibular organs or rather reflect local damage to the vestibular hair cells. Central to this question is the fact that vascular supply and innervation are provided by two separate branches of the vestibular artery and nerve, respectively. While the superior branch of the vestibular nerve and artery supports the anterior and horizontal SCC and the utriculus, the inferior branch is linked to the posterior SCC and the sacculus. In each patient, we categorized anterior, posterior and horizontal SCC function and oVEMPs/cVEMP as “bilaterally normal,” “unilaterally reduced,” or “bilaterally reduced.” We then correlated otolith and SSC function according to the anatomy as described above (i.e., oVEMPs vs. anterior SCC function, oVEMPs vs. horizontal SCC function, cVEMPs vs. posterior SCC function). This segregation showed that a corresponding pattern of SCC and otolith hypofunction was present in 60.4% (oVEMPs and horizontal SCCs), 34.7% (oVEMPs and anterior SCCs), and 48.5% (cVEMPs and posterior SCCs) of cases (see Table 7 for details). Specifically, utricular and SCC function were corresponding (i.e., either both normal or both abnormal) with a significantly higher rate for the horizontal canal compared with the anterior canal (*p* < 0.001).

**Table 7:** Comparison of semicircular canal (SCC) and otolith function according to the vestibular anatomy—percentage of cases that match in function for various disorders.

Disorders	Hor SCCs vs. utriculus	Ant SCCs vs. utriculus	Post SCC vs. sacculus
Unknown cause (*n* = 54)	32/54 (59.3%)	18/54 (33.3%)	16/54 (29.6%)
Vestibulotoxic drugs (*n* = 12)	8/12 (66.7%)	3/12 (25.0%)	10/12 (83.3%)
Menière’s disease (*n* = 11)	6/11 (54.5%)	4/11 (36.4%)	6/11 (54.5%)
Various causes (*n* = 17)	9/17 (52.9%)	2/17 (11.8%)	9/17 (52.9%)
Infection-related bilateral vestibular loss (*n* = 7)	6/7 (85.7%)	6/7 (85.7%)	5/7 (71.4%)
All pooled (*n* = 101)	61/101 (60.4%)	35/101 (34.7%)	49/101 (48.5%)

Noteworthy, in those 83 patients with bilateral hypofunction of the posterior SCCs, saccular function was preserved bilaterally (*n* = 20) or unilaterally (*n* = 20) in 40, showing a discrepancy between canal and otolith function despite common innervation/vascular supply by the inferior branch of the vestibular nerve/vestibular artery. Discrepancies between SCC and otolith function showed distinct patterns: bilaterally (*n* = 4) or unilaterally (*n* = 19) preserved utricular function was noted in 73 patients with bilateral hypofunction of the horizontal canals, while bilaterally (*n* = 26) or unilaterally (*n* = 16) impaired utricular function was noted in 53 patients with bilaterally normal function of the anterior canals. While we found no cases with bilaterally normal oVEMPs and bilateral hypofunction of the anterior canals, we identified 42 patients with unilaterally or bilaterally abnormal oVEMPs and bilaterally normal function of the anterior SCCs, showing a dissociation between anterior-canal function and utricular function. For the horizontal SCCs, we observed six cases with (unilaterally) impaired oVEMPs and bilaterally normal canal function, while oVEMPs were bilaterally normal in seven patients with bilateral (*n* = 4) or unilateral (*n* = 3) hypofunction of the horizontal SCCs, suggesting minor discrepancy only between these two parameters. Segregating this analysis for the previously specified disorders, we found the rate of corresponding canal and otolith function to vary between underlying disorders and pairs of canal/otolith function—for details see Table 8.

**Table 8:** All bilateral vestibular loss (BVL) cases with vHIT, ocular vestibular-evoked myogenic potentials (oVEMPs) and cervical vestibular-evoked myogenic potentials (cVEMPs) (*n* = 101).

		Hor semicircular canal (SCC) function				Ant SCC function				Post SCC function		
						
		Normal	Unilat red.	Bilat red.		Normal	Unilat red.	Bilat red.		Normal	Unilat red.	Bilat red.
**All groups pooled^a^**												

oVEMPs	Normal B	6	3	4	oVEMPs	11	2	0	cVEMPs	0	3	20
	Abnormal U	4	5 (2)	19		16	7	7		2	6	20
	Abnormal B	2	6	50		26	15	17		2	5	43

**Subgroup analyses^a^**												

**BVL of unknown cause (*n* =54)**												
oVEMPs	Normal B	4	3	2	oVEMPs	8	1		cVEMPs		3	15
	Abnormal U	2	2 (1)	10		10	2 (1)	2			2 (3)	14
	Abnormal B		4	26		14	8	8		2	1	14

**Aminoglycoside-induced vestibulotoxicity (*n* = 12)**												
oVEMPs	Normal B				oVEMPs				cVEMPs			1
	Abnormal U			2		1	1					1
	Abnormal B		2	8		5	3	2				10

**Menière’s disease (*n* = 11)**												
oVEMPs	Normal B	2		1	oVEMPs	3			cVEMPs			2
	Abnormal U	1	1	2		2	1	1		1	1	
	Abnormal B	1		3		4					2	5

**Infectious inner-ear disorders (*n* = 7)**												
oVEMPs	Normal B				oVEMPs				cVEMPs			
	Abnormal U		1	1		1	1					2
	Abnormal B			5				5				5

**Various disorders (*n* = 17)**												
oVEMPs	Normal B			1	oVEMPs		1		cVEMPs			2
	Abnormal U	1	1 (1)	4		2	0 (1)	4		1		3
	Abnormal B	1		8		3	4	2			2	9

*^a^All patients with corresponding impairment of both sensors compared are reported in the grey-shaded areas. Note that values in brackets indicate cases with unilateral impairment on non-corresponding sides (e.g., left-sided utricular hypofunction and right-sided horizontal canal impairment)*.

**Figure 5:**
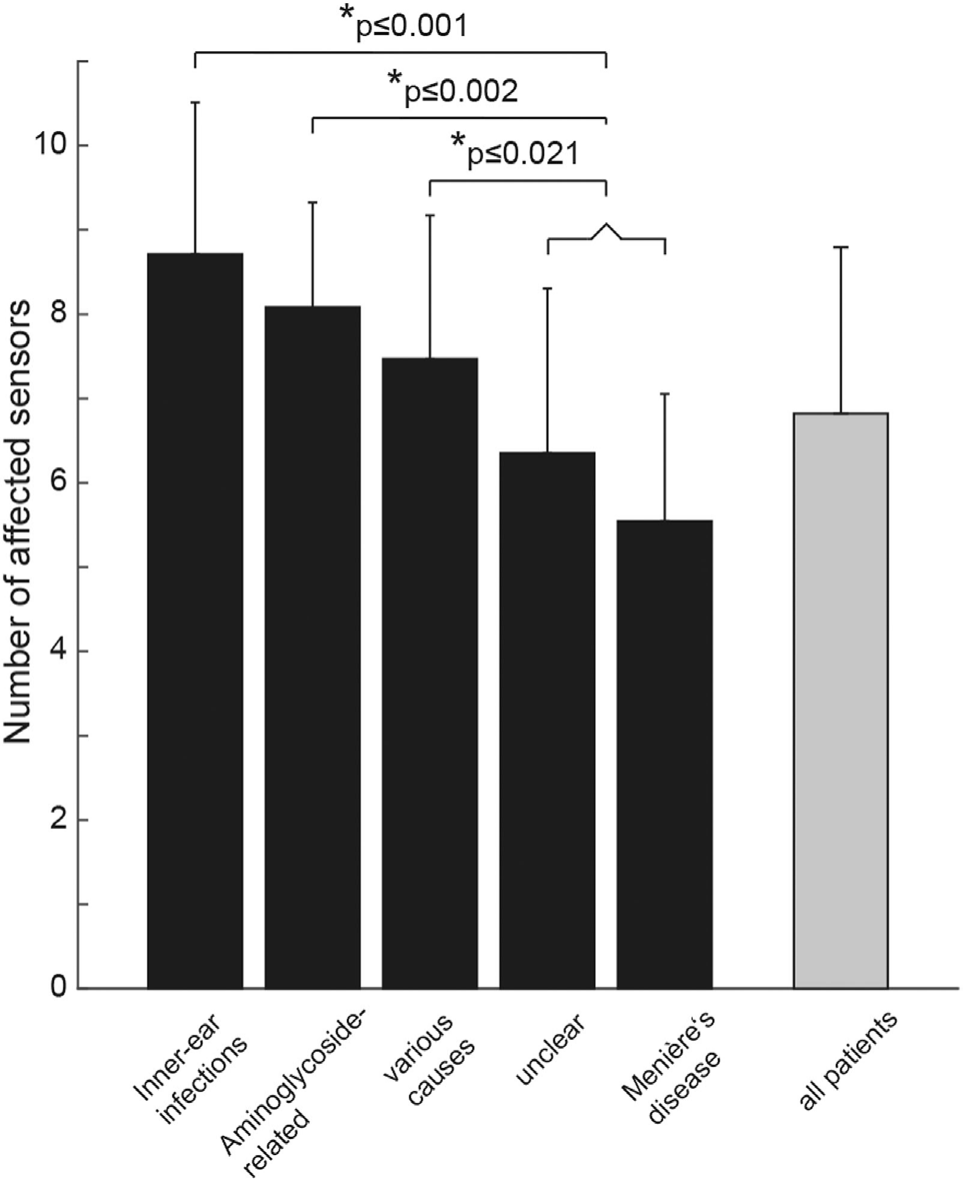
Bar plot illustrating the mean (±1 SD) number of affected vestibular sensors (for each side: anterior/posterior/horizontal semicircular canal, utriculus, sacculus) separately for the different subgroups (in black) and for all patients pooled (in gray). “Various” includes patients with trauma (*n* = 2), bilateral sensorineural hearing loss (*n* = 3), bilateral schwannoma (*n* = 4), combined schwannoma and vestibular neuropathy (*n* = 1), cerebellar ataxia, neuropathy, vestibular areflexia syndrome (*n* = 2), autoimmune (*n* = 3), and central causes (*n* = 2). Statistically significant differences (SPSS: generalized linear model) are indicated by an asterisk (*).

The average (±1 SD) number of damaged sensors (from 2 in case of isolated involvement of 2 canals to 10 in case of complete bilateral vestibular loss) in the entire study population was 6.8 ± 2.0. We noted a significant main effect of the underlying diagnosis (df = 4, chi-square = 26.650, *p* < 0.001; a generalized linear model used as data was normally distributed). Pairwise comparisons demonstrated significantly (*p* ≤ 0.001) higher numbers of affected sensors for patients with BVD related to infectious disorders than for those with BVD linked to Menière’s disease and unclear causes. Compared with patients with BVD related to Menière’s disease, patients with aminoglycoside-related BVD showed involvement of significantly more sensors (*p* < 0.001) (see Figure 5 for detailed statistics).

### Hierarchical Cluster Analysis

**Figure 6:**
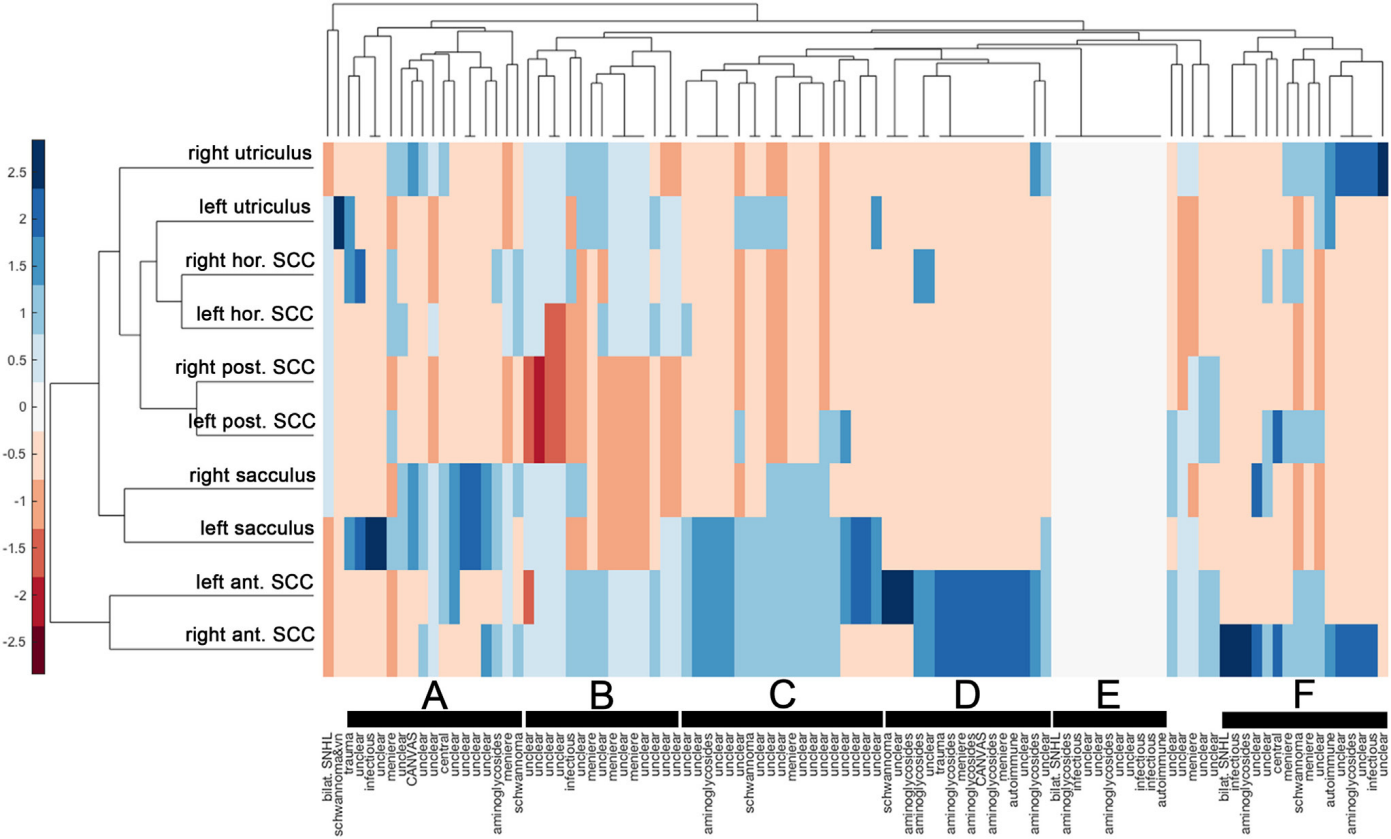
Hierarchical cluster analysis based on the overall rating (intact vs. impaired) of each vestibular end organ. A heat map indicates the functional state [from intact (=blue) to deficient (=red)] for each vestibular end organ (*n* = 10) and patient (*n* = 101). The heat map was clustered by Euclidean distance. The data were standardized along the columns of data, i.e., for the individual results from single subjects, resulting in a range of colors between 2.5 (blue) to −2.5 (red) as indicated by the legend on the left side. Diagnosis in each patient is provided along the *x*-axis. Cluster dendrograms indicate those patients (*x*-axis) and vestibular end organs (*y*-axis) that are the least different, as these groups cluster together first. Cluster analysis identified 5 clusters with at least 15 patients (nodes A, B, C, D, and F) and one smaller cluster (node E), which are all marked with black bars.

Cluster analysis resulted in a heat map with the different vestibular end organs (*n* = 10 in total) and patients (*n* = 101) assessed separately (Figure 6). The dendrogram illustrating the clustering of the vestibular sensors (at the left border of the figure) indicated that identical sensors on the left side and the right side merged first (this was true for all three pairs of canals (anterior, posterior, horizontal) and the saccular organs). Only the two utricular organs merged later. Mergers between the different vestibular end organs occurred first for the posterior canals and the horizontal canals (that already had merged with the left utriculus) and then with the right utriculus. At the next higher level of merger, the saccular organs were added, whereas the anterior canals were added last. The top dendrogram [showing the clustering of the patients (columns)] indicated five clusters with at least 15 nodes (marked with black bars). The distinguishing feature in node A (*n* = 17, 8/17 diagnosed as “unclear”) was the state of the saccular organs (being spared whereas all other sensors were impaired), whereas in node B (*n* = 15, 11/15 rated as “unclear”) it was the state of the saccular organs and the posterior SCCs (both being impaired, whereas all other sensors were relatively spared). This pattern (node B) is consistent with the innervation/vascular supply by the inferior branch of the vestibular nerve. In contrast, for node C, the state of the anterior canals and the saccular organs were the distinguishing features (both remaining intact), whereas for node D it was the residual functioning of the anterior canals that mattered. While most patients belonging to node C remain undiagnosed (“unclear” in 16/19), a significant fraction of patients in node D received a diagnosis of aminoglycoside-related bilateral vestibulopathy (6/16). In node F (*n* = 16), patterns were variable, whereas for some patients only a single anterior canal was outstanding (i.e., being functionally intact), others showed a more balanced pattern with grouping of, e.g., the anterior canals, the posterior canals and the utricular organs. In a smaller node (node E, *n* = 11) with no distinguishing features on the heat map (i.e., white (= 0) on the color bar) all patients with impairment of all 10 vestibular end organs were included. In this cluster, diagnoses as infectious inner-ear disease (*n* = 3), bilateral SNHL (*n* = 1) and autoimmune disorder (*n* = 1) were overrepresented.

## Discussion

Bilateral vestibular loss of function is often subtle initially and when eventually diagnosed remains of unknown origin in 20–50% of all cases (7, 30–33). Previously, we provided a detailed characterization of horizontal and vertical canal function in BVL, reporting disease-specific patterns of SCC hypofunction which may help in the differential diagnosis and allow more specific treatment (7). Particularly, we described relative sparing of anterior-canal function in aminoglycoside-related BVL and bilateral Menière’s disease, while in BVL secondary to inner-ear infections or early hearing loss all SCCs were affected with similar frequency. Here we provide a detailed peripheral-vestibular mapping with hierarchical clustering of quantitative canal and otolith testing in 101 BVL patients asking which pathomechanisms best reflect the patterns of vestibular end-organ damage observed and whether these patterns are disease-specific. The combination of SCC and otolith damage observed in our BVL patients was variable and matched a vascular/neuronal pattern only in about half the cases, limiting the role of this pathomechanism for the development of the pattern of peripheral-vestibular hypofunction in BVL. Rather, the observed dissociation of horizontal and anterior-canal function in the presence of utricular hypofunction suggest that damage to the specific vestibular sensors (i.e., the hair cells) plays an important role.

### The Value of Hierarchical Cluster Analysis in Pattern Recognition in BVL

The implementation of hierarchical cluster analysis in the assessment of SCC and otolith damage in BVL patients proved very useful and confirmed and extended our observations from previous work (7). Specifically, hierarchical cluster analysis indicated that identical sensors on the left side and the right side merged first (with the exception of the utricular organs). This observation suggests that in patients with bilateral vestibulopathy the individual vestibular end organs are typically in a similar condition in both labyrinths. The observation that the anterior canals merged latest suggests that the condition of the anterior canals is a distinguishing feature in our patients. Furthermore, cluster analysis revealed several nodes with different patterns of vestibular end-organ impairment, ranging from isolated impairment of the posterior canals to sparing of the anterior canals and/or the saccular organs to loss of function of all vestibular end organs. The cluster analysis therefore further emphasizes the broad spectrum of SCC and otolith impairment in patients with BVL. At the same time, certain diagnoses were enriched in single nodes. Most consistent was the accumulation of patients with aminoglycoside-related vestibulotoxicity in the node with anterior-canal sparing, supporting our findings from the statistical analysis.

### Otolith Function—Correlations and Comparison With the Literature

Since we included only patients with bilateral SCC hypofunction, the lower rate of utricular/saccular hypofunction compared with SCC hypofunction is likely due to a selection bias. With utricular (87.1%) and saccular (78.2%) hypofunction occurring at similar frequencies, our findings emphasize the frequent involvement of the otolith organs and confirm a previous report that stated comparable rates of utricular (64%) and saccular (61%) deficits (13). Whereas rates of impairment were somewhat larger in our case series compared with Agrawal and colleagues, this was most likely due to differences in the methodology: besides response amplitudes [as applied by Agrawal et al. (13)], we took latencies and asymmetry ratios into account and assessed vertical canal function also. Therefore, bilateral horizontal SCC hypofunction was not a prerequisite, as in studies that relied on caloric irrigation for diagnosing BVL. In another study, using bilateral horizontal SCC hypofunction as the inclusion criteria, cVEMPs amplitudes were abnormal (bilateral: *n* = 51, unilateral: *n* = 8) in 70.2% of patients (12), which is in a similar range as the 76.7% reported here in those 73 patients with bilateral horizontal SCC hypofunction. Nonetheless, Zingler and coworkers proposed relative sparing of saccular function, as compared with 40 patients with bilaterally absent response on caloric irrigation, cVEMPs were unilaterally absent only in four patients and bilaterally absent in none (12). In our study, cVEMPs were absent in 30 cases (bilateral = 20; unilateral = 10), representing a clearly higher frequency of absent saccular function than proposed by Zingler and colleagues. Sparing of saccular function was also reported in a series of five patients with BVL on caloric irrigation but preserved cVEMPs (34). However, the true rate of saccular impairment in BVL remains to be determined, as requiring bilateral (horizontal) SCC hypofunction as inclusion criterion results in a selection bias.

Rates of abnormal utricular function were disease-dependent, showing significantly larger proportions of impairment in those disorders that resulted in the most extensive SCC damage, i.e., BVL related to inner-ear infections and aminoglycoside-toxicity. Specifically, rates of abnormal oVEMPs were higher in patients with aminoglycoside-related BVL compared with patients with bilateral Menière’s disease (91.7 vs. 54.6%, *p* = 0.039), while no such difference was found for cVEMPs. Therefore, oVEMPs may be helpful in the distinction between BVL related to aminoglycoside-toxicity and Menière’s disease. This is consistent with findings from Ref. (13). Noteworthy, a pattern of bilaterally impaired utricular function and bilaterally preserved anterior-canal function was noted only in 5/12 patients diagnosed with aminoglycoside-related bilateral vestibulopathy (sensitivity = 41.7%, specificity = 76.4%). This underlines that also for these patients, the pattern of impairment can be variable, with, e.g., only one anterior canal spared or only one utricle affected.

Furthermore, bilaterally impaired utricular function was a very good predictor for bilateral loss of function of the horizontal canals, as in 50 out of 58 cases with bilateral utricular impairment bilateral horizontal canal hypofunction was present as well. Likewise, bilateral impairment of saccular function predicted with high probability bilateral loss of function of the posterior canals (bilateral posterior-canal hypofunction was found in 43 out of 50 cases with bilateral saccular impairment). Bilateral anterior-canal hypofunction, on the other hand, was found in only 17/58 cases with bilateral utricular loss of function, making the utricles a poor predictor for anterior-canal function.

### Correlating SCC and Otolith Function in the Entire Study Population and in Disease-Specific Subgroups

Complete bilateral loss of peripheral-vestibular function (i.e., hypofunction of all six SCCs, both utricles and saccules) was present in 11 out of 101 patients (10.9%). Much more frequently, loss of function was restricted to a part of all vestibular end organs. Specifically, we found no patients with bilaterally normal oVEMPs and bilateral hypofunction of the anterior canals. In contrast, we identified 42 cases with unilaterally or bilaterally abnormal oVEMPs and bilaterally normal function of the anterior SCCs. This shows that anterior-canal hypofunction is usually accompanied by abnormal oVEMPs, while loss of utricular function may be isolated, i.e., not reflected in anterior-canal hypofunction. For the horizontal SCCs, we observed only six cases with (unilaterally) abnormal oVEMPs and bilaterally normal horizontal canal function, while oVEMPs were bilaterally normal in seven patients with bilateral or unilateral hypofunction of the horizontal SCCs. This indicates that utricular hypofunction and hypofunction of the horizontal SCCs are frequently linked—a pattern that is also demonstrated in the dendrogram (**Figure 6**). For the posterior SCCs, we observed only four cases with (unilaterally) abnormal cVEMPs and bilaterally normal canal function, while cVEMPs were bilaterally normal in 23 patients with bilateral (*n* = 20) or unilateral (*n* = 3) hypofunction of the posterior SCCs. This suggests that posterior-canal hypofunction can be isolated, while cVEMP abnormalities are usually accompanied by posterior-canal hypofunction.

Consistent patterns between (1) posterior SCC and saccular function, (2) horizontal SCC and utricular function, and (3) anterior SCC and utricular function were noted with rates of 49, 60, and 35%, respectively, only. This finding shows that canal and otolith function in BVL is often dissociated. Noteworthy, the pattern varied for different disorders, most likely linked to the underlying pathomechanisms. For disorders with diffuse peripheral-vestibular damage, such as infectious inner-ear disease, rates of concomitance were 72% or higher, while in other disorders such as Menière’s disease rates were between 36 and 55% for the different combinations. Similarly, in the largest subgroup (idiopathic BVL), rates were between 30 and 59%. These fairly low percentages speak against combined damage according to the vascular supply or the innervation of the vestibular organs as a common cause in BVL. Rather, the patterns observed in our study favor other, likely local mechanisms affecting the hair cells of the distinct vestibular end organs including inflammation/infection, toxins, and endolymphatic hydrops. Sparing of certain vestibular end organs (as the anterior canals) may point to varying susceptibility to these mechanisms.

In a subgroup of patients (*n* = 12), the horizontal canals were bilaterally spared when assessed by vHIT, whereas both posterior canals were abnormal in 10 out of these patients and the anterior canals were impaired in only four cases. This pattern may suggest a sequential loss of function of the different SCCs, with posterior-canal function showing impairment first, as discussed also by the authors in a previous publication (35). Furthermore, these 12 patients had higher rates of abnormal cVEMPs than oVEMPs (75 vs. 50%), suggesting a predominantly inferior vestibular branch/artery involvement. Interestingly, this pattern was mostly associated with BVL of unclear origin and Menière’s disease. To determine whether these patients would have been diagnosed as having BVL when testing the horizontal canals only by use of caloric irrigation, we retrieved results on caloric irrigation as well in these 12 cases. For diagnosing bilateral hypofunction on caloric irrigation, a nystagmus with a mean peak slow-phase velocity of <5°/s for cold- and warm-water irrigation on both sides was required (31). Noteworthy, only two of those 12 patients met criteria for BVL on caloric irrigation, whereas 6 demonstrated unilateral hypofunction only and four had bilaterally normal responses. Therefore, 10 of those patients would have been missed when using caloric irrigation only for diagnosing BVL. Previously, the combination of bilaterally absent cVEMPs and bilaterally normal response on caloric irrigation has been reported in three patients with BVL of unclear origin selected from a large sample of 1,025 patients presenting to a specialized dizzy clinic (36). Furthermore, Fujimoto and colleagues reported dissociated BVL (e.g., abnormal caloric irrigation on one side and impaired cVEMPs on the other side) in 20.3% of their BVL patients (37), again emphasizing the varying combination of involvement of the different vestibular sensors.

Overall, the extent of SCC and otolith damage (as reflected by the number of vestibular sensors affected) was disease-dependent, showing significantly higher rates for BVL related to inner-ear infections and aminoglycoside-toxicity than for Menière’s disease. This further stresses out differences in the underlying pathomechanisms leading to BVL and available residual vestibular function. The importance of otolith testing was previously emphasized by others as well: Agrawal and coworkers have reported that otolith dysfunction had a greater association with functional impairment (as assessed by the dizziness handicap inventory) compared with SCC function (13) and Lempert and colleagues have demonstrated a functional role of the otolith-ocular reflex in visual stabilization during high frequency linear head motion (9). Thus, loss of otolith function may be reflected in more severe symptoms and therefore, from a therapeutic perspective, physical therapy and balance training should especially be enforced in those patients with aminoglycoside-related BVL and infectious inner-ear disorders.

### Limitations

Our study design was retrospective and patient selection depended on a single test (vHIT). Therefore, these patients did not receive a standardized clinical neuro-otologic examination and there were no prospectively defined diagnostic criteria for specific disorders. For Menière’s disease, guidelines according to the AAO-HNS from 1995 were used and MR imaging was mandatory for cases with vestibular schwannoma. The diagnosis of aminoglycoside-related vestibulopathy and BVL due to inner-ear infection was based on the patient’s medical files. Furthermore, in half of our patients the underlying cause of disease could not be identified. This is within the range of 20–51% of cases with an idiopathic origin reported previously (30, 31, 33, 38). Noteworthy, as bilateral SCC hypofunction was a prerequisite for inclusion, we may have missed patients with isolated bilateral loss of saccular function (36).

While for the vHIT gain values have been shown to remain stable in healthy human subjects until very high age (no significant changes until ages 80–89 years) (39), significantly higher rates of absent responses were observed at advanced age for ocular (≥80 years) and cervical (≥70 years) VEMPs (40). The same authors noted decreases in peak-to-peak response amplitude in cVEMPs and oVEMPs with age and an increase in n10 latency (oVEMPs) by 0.12 ms per decade, while no such increase was noted for p13 latencies (cVEMPs) (40). With regards to our study, 24 patients were aged between 70 and 80 years, and 14 patients were aged more than 80 years. We therefore cannot exclude that some abnormalities in our very old patients were physiological and that we therefore may have overestimated the extent of otolith damage in these patients. Noteworthy, diagnosis of BVL did not depend solely on otolith function in our study but required bilaterally abnormal SCC function as well.

Currently, there is ongoing controversy about the diagnostic criteria for BVL. While recently proposed diagnostic criteria by the classification committee of the Bárány Society propose impairment of both horizontal SCCs (6), we have also included patients that presented with bilateral impairment of the vertical canals only or suffered from a combination of horizontal and vertical canal impairment. While this may result in an overall less affected patient population, this reflects a real-life scenario, with only few patients showing completely abolished vestibular function bilaterally.

## Conclusion

Detailed peripheral-vestibular mapping with hierarchical clustering in BVL revealed a patchy loss of function of the SCCs and the otolith organs in the majority of cases, which could be segregated in six nodes with distinct patterns of SCC and otolith impairment. Our findings therefore confirm and extend the previous notion of variable and at least partially disease-specific loss of SCC function in BVL. Utricular and saccular impairment was frequent and occurred in similar fractions. Overall, bilaterally impaired utricular function was a very good predictor for bilaterally deficient horizontal canals and bilateral saccular impairment predicted bilateral posterior-canal damage with high probability. In contrast, bilateral utricular hypofunction was a poor predictor of anterior-canal dysfunction. The extent of SCC and otolith impairment was disease-dependent, showing most extensive damage in BVL related to inner-ear infection and aminoglycoside exposure and more selective impairment in other groups such as BVL secondary to Menière’s disease. Specifically, assessing utricular function may help in the distinction between aminoglycoside-related BVL and bilateral Menière’s disease. Based on these disease-specific patterns of SCC and otolith function in BVL, we promote complete peripheral-vestibular mapping, since this may be useful in the differential diagnosis and eventually treatment decisions in BVL of presumably unknown origin.

## Ethics Statement

This study was carried out in accordance with the recommendations of the Cantonal Ethics Committee Zurich and in accordance with the Declaration of Helsinki. As this was a retrospective database analysis, retrieval of informed written consent from all involved patients was not feasible. The protocol was approved by the Cantonal Ethics Committee Zurich and exempt for retrieval of written informed consent was granted (study protocol 2013-0468).

## Author Contributions

AT drafted the manuscript, analyzed the data, and conceived of the study. CB helped in the data analysis and interpretation. EB performed the data collection. KW was involved in the design of the study, participated in the data analysis and the statistical analysis, and critically reviewed and edited the manuscript. All the authors read, revised, and approved the final version of the manuscript.

## Conflict of Interest Statement

AT, CB, and EB declare that they have no conflict of interest. KW acts as an unpaid consultant and has received funding for travel from Otometrics.
